# Getting resources to those who need them: the evidence we need to budget for underserved populations in sub‐Saharan Africa

**DOI:** 10.1002/jia2.25707

**Published:** 2021-06-30

**Authors:** Lawrence C Long, Sydney Rosen, Brooke Nichols, Bruce A Larson, Nhlanhla Ndlovu, Gesine Meyer‐Rath

**Affiliations:** ^1^ Department of Global Health School of Public Health Boston University Boston MA USA; ^2^ Department of Internal Medicine School of Clinical Medicine Faculty of Health Sciences University of Witwatersrand Johannesburg South Africa; ^3^ Health Economics and Epidemiology Research Office Wits Health Consortium Johannesburg South Africa; ^4^ Centre for Economic Governance and Accountability in Africa (CEGAA) Pietermaritzburg South Africa

**Keywords:** vulnerable populations, HIV infections, cost, budgets, resource allocation, Africa

## Abstract

**Introduction:**

In recent years, many countries have adopted evidence‐based budgeting (EBB) to encourage the best use of limited and decreasing HIV resources. The lack of data and evidence for hard to reach, marginalized and vulnerable populations could cause EBB to further disadvantage those who are already underserved and who carry a disproportionate HIV burden (USDB). We outline the critical data required to use EBB to support USDB people in the context of the generalized epidemics of sub‐Saharan Africa (SSA).

**Discussion:**

To be considered in an EBB cycle, an intervention needs at a minimum to have an estimate of a) the average cost, typically per recipient of the intervention; b) the effectiveness of the intervention and c) the size of the intervention target population. The methods commonly used for general populations are not sufficient for generating valid estimates for USDB populations. USDB populations may require additional resources to learn about, access, and/or successfully participate in an intervention, increasing the cost per recipient. USDB populations may experience different health outcomes and/or other benefits than in general populations, influencing the effectiveness of the interventions. Finally, USDB population size estimation is critical for accurate programming but is difficult to obtain with almost no national estimates for countries in SSA. We explain these limitations and make recommendations for addressing them.

**Conclusions:**

EBB is a strong tool to achieve efficient allocation of resources, but in SSA the evidence necessary for USDB populations may be lacking. Rather than excluding USDB populations from the budgeting process, more should be invested in understanding the needs of these populations.

## 1 INTRODUCTION

Many countries in sub‐Saharan Africa (SSA) are characterized as having hyperendemic HIV epidemics in large parts of the population who are at low to moderate risk of HIV while simultaneously experiencing concentrated sub‐epidemics in populations who are at high risk of HIV acquisition or transmission and for whom no sampling frame exists and acknowledging membership may be risky [1, 2, 3]. To date, the HIV response in SSA has focused largely on those who are at relatively low to moderate risk of HIV acquisition, but account for the majority of new infections because of the relative size of the population. The large size of these populations has typically made them easier to reach than those at highest risk, who may not be easy to identify or even enumerate. This has resulted in significant progress in achieving the 90‐90‐90 targets overall but does not necessarily reflect equal progress within the concentrated sub‐epidemics [4, 5, 6]. Since these sub‐epidemics have been estimated to contribute disproportionately to onward infections, lower coverage or service effectiveness within these subgroups may hamper epidemic control [2, 7, 8, 9].

Even in eastern and southern Africa, where the majority of transmission is occurring in a large population at relatively low risk of HIV acquisition, infections related to the populations at increased risk of HIV, such as injecting drug users, sex workers, men who have sex with men (MSM), prisoners and transgender people, and their partners accounted for almost a third of new infections (28%) in 2019 [10, 11]. Despite the increased HIV burden these populations face and their reported disproportionately high contribution to onward HIV transmission, relatively small shares of HIV budgets and programming were historically targeted to them [12, 13]. More recently some donors have earmarked funds for certain populations considered to be at increased risk such as DREAMS and Key Population Investment Fund [14, 15]. In addition, traditional “key” population programming focuses broadly on reaching the entire population, rather than just the underserved individuals among them. Within each any population affected by HIV, there are those who face the triple burden of high HIV transmission risk, low access to services and social stigma assigned to their risk group. Ignoring those who are underserved and carry a disproportionate HIV burden (USDB) will limit our ability to achieve epidemic control.

Reaching this USDB population, important as it may be, has been increasingly complicated by resource constraints. Over the last decade, global funding for HIV has remained relatively constant, whereas the number of people on treatment in many countries, in particular in SSA, has increased [16, 17, 18]. As a result, the proportion of budgets allocated to HIV treatment is growing, whereas investment in other areas declines [17]. Allocating funds for the difficult work of identifying, reaching and supporting USDB populations is more difficult than ever given these constraints, in particular in countries where the HIV response is focused on the large population who are at low to moderate risk.

In order to encourage the best use of limited and decreasing HIV resources, evidence‐based budgeting (EBB) has been gradually adopted by both donors and governments for HIV programming [19, 20, 21, 22, 23, 24, 25]. For many countries in SSA donor funding, which leans heavily on EBB practices, accounts for the majority of HIV programming [26]. Evidence‐based budgeting has many forms but refers broadly to the use of empirical data to justify the allocation of financial, human and other resources to achieve measurable targets. The principle of EBB is premised on the availability of data to inform this process; lack of or inaccurate data may result in inappropriate budgeting [27]. If data availability and research‐based evidence were equal across all interventions and populations, EBB would result in an efficient allocation of resources, but the data and evidence necessary to support interventions for hard to reach, marginalized and vulnerable populations are scarce [11]. Donors and governments applying EBB, where there are evidence gaps, potentially further disadvantages USDB populations.

In this article, we outline the critical data required to use EBB to support USDB people in the context of the SSA epidemics. We use specific examples from South Africa to illustrate the potential challenges and gaps that policy makers and researchers must fill to support USDB interventions going forward.

## 2 DISCUSSION

### 2.1 Overview

Evidence‐based budgeting aims to replace incremental budgets which are based on last year’s budget with small increases, to a rationalist theory that adds an aspect of performance or outcome measurement [28]. In this paper, we focus on the critical evidence required for an intervention to be considered in an EBB budget allocation decision in the context of USDB populations.

To be considered in an EBB cycle, an intervention needs at a minimum to have an estimate of a) the average cost, or cost per recipient of the intervention; b) the effectiveness of the intervention in achieving its stated goal and c) the size of the intervention target population. Below we briefly discuss each of these and explain why methods commonly used are not sufficient for generating valid estimates for USDB populations.

**Figure 1:**
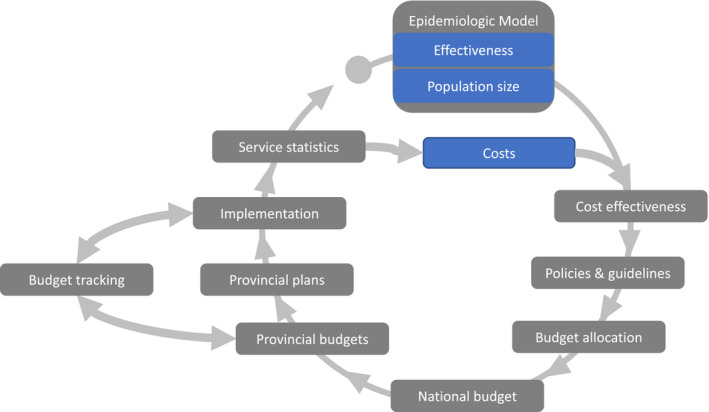
Stylized budgeting and planning process for HIV in South Africa. Critical data highlighted in blue. Adapted from Murphy et al. [22] and Ndlovu [23].

***Example: Evidence‐based budgeting in South Africa***. The full budget process for a country is complicated and unique. Figure 1 shows a stylized version of the South African national and provincial HIV budgeting and planning process only and highlights how cost, effectiveness and population size data are critical in order for an intervention to be considered for inclusion during budget allocation [29, 30].

### 2.2 Cost estimation

Broadly speaking, we estimate the cost of an intervention by multiplying the quantities of resources needed to implement the intervention by their price [31]. Methods can be stratified by how resources are identified (gross vs. micro) and valued (top down vs. bottom up; perspective of the provider or society) [32]. Bottom‐up, micro‐costing is often considered the most accurate method as it allows the identification of all cost components at the individual level, but micro‐costing poses challenges for novel interventions aimed at reaching USDB populations, for several reasons.

First, in countries without USDB interventions, costs cannot be estimated based on existing programmes, and a “normative best practice” approach is taken [33]. Normative, bottom‐up costing starts with guidelines and converts them into resource usage and costs. To generate accurate costs, the guidelines need to not only clearly indicate all the resources required, but also how they are to be utilized to achieve the desired outcome‐ and the intervention, when implemented, must have high fidelity to the guidelines. Guidelines are however often agnostic of population. For example while the clinical management of pre‐exposure prophylaxis (PrEP) for a man is relatively standardized [34], the additional services necessary to achieve a good clinical outcome may differ by population (i.e. a man in a sero‐discordant relationship with a woman vs. a man who is having unprotected anal sex with a man). As a result, normative costing often misses essential resources needed to make programmes effective for marginalized populations.

The second challenge with using micro‐costing for USDB interventions is that, even once programmes are implemented, routine bottom‐up micro‐costing requires records that accurately capture all the resources necessary to achieve the desired health outcome. Routine clinical records typically capture medication, laboratory tests and visits reasonably well. Outside of prospective research settings, clinical records are not good at capturing how the patient came to present at the facility (i.e. outreach programme, communication strategy), or whether they receive ancillary services (i.e. counselling, sexually transmitted infection (STI) screening) or further support to remain engaged in care (i.e. support groups). Interventions targeting USDB populations often require services in addition to standard routine treatment in order to access, link and retain these patients in care. These services (many of which may take place outside of the facility) require additional resources and are integral to the programme. Initially, such data will likely need to come from pilot programmes or clinical trials.

***Example: Change to average costs for services targeted at USDB populations in South Africa***. In our review of cost estimates for the South African HIV Investment Case, we found that the cost of activities that specifically focused on reaching USDB populations such as condom distribution outside standard channels (i.e. through brothels, schools or hotels) and female sex worker (FSW) HIV testing had higher average costs compared to the same services focused on populations at low to moderate risk and easier to reach. Targeting these groups also often had a higher impact, yielding variable cost‐effectiveness [35], which highlights the importance of programme costing specific to the population it serves.

### 2.3 Effectiveness estimation

There are traditionally two approaches to defining the effectiveness of an intervention; 1) effectiveness based on clinically‐relevant outcome measures (i.e. patients diagnosed, patients virally suppressed) or 2) effectiveness based on synthetic measures of health gain (i.e. cases averted, disability‐adjusted life years averted, life years saved) [31]. HIV programmes gravitate towards these clinical outcome measures (such as 95‐95‐95 [6]) because they are easy to measure and seem objective. Clinical outcomes may be useful and appropriate when the following two conditions hold: a) there is a constrained choice set of interventions addressing the same issue (i.e. interventions aimed at increasing HIV testing yield in men), and b) outcomes are final endpoints or good predictors of the final intended outcome (i.e. suppressed viral load at 12 months predicting life years saved) [36].

Using clinical outcomes to measure effectiveness is problematic when applied to USDB populations because one or both of these conditions might not hold. For health budgeting at the national level, it is likely that policy makers are being asked to weigh choices across interventions targeting many different populations with different outcomes. Constrained choice set decision‐making (i.e. the feasible set of options is limited) may however apply in donor programmes that are specific to achieving a certain outcome in a specified population (i.e. a priori defined budget for testing injecting drug users) and allow for using a clinical outcome.

Additionally, interventions which achieve the same interim clinical or natural outcomes can have different final outcomes in USDB populations compared to a population at low to moderate risk (i.e. a female sex worker with a suppressed viral load at 12 months is likely to generate more life years saved through prevented infections than a woman of the same age with a single sexual partner). While requiring the use of mathematical models and thus being more complicated to calculate and report for national programmes, generic measures of health gains (i.e. infections averted, life years saved) are more likely to capture the full health benefit associated with reaching USDB populations to allow a fair comparison across interventions.

***Example: Impact of adding effectiveness to decision making for PrEP in South Africa***. In a recent cost‐effectiveness analysis of PrEP provision to different target populations in South Africa, we found that, although the cost of PrEP was very similar between all target groups, adding effectiveness (cost per HIV infection averted) showed that provision to MSM and sex‐workers would be more cost effective than to adolescents of both genders, young women and men, or pregnant women, regardless of successful self‐targeting of these groups by risk behavior – with the exception of high‐risk female adolescents [37].

### 2.4 Population estimation

One of the most challenging data gaps are accurate estimates of USDB population size; UNAIDS reported only four population size estimates for those at increased risk (all prison populations) for eastern and southern Africa in 2018/2019 [11]. The World Health Organization (WHO) and UNAIDS have issued guidelines on how best to approach this topic for different purposes; for budgeting, crude national estimates might be appropriate, but in order to prioritize and set targets, localized estimates become necessary [38, 39]. In SSA, USDB populations are unlikely to be captured accurately in traditional national surveillance tools or routine clinical data systems as a result of the stigma and, in some instances, criminalization [40, 41]. As a result, there have been a number of innovative approaches put forward that allow national population estimates for those at increased risk to be extrapolated from local estimates [42]. A review of the methods used in low‐ and middle‐income countries found that the most popular approaches to estimating populations at increased risk were multiplier, capture‐recapture, census and enumeration and programmatic mapping [43]. The challenge with these approaches is that the estimates are sensitive to the methods used and can result in variation across estimates, which may raise questions regarding their validity for policy making [44, 45, 46]. To address this, it may be appropriate to apply multiple estimation methods to provide a range around USDB population estimates [43]. Recent evidence also suggests that even when population estimates are available in SSA, there is little evidence that they are being used to inform policy decisions including resource allocation [47]. These estimates are critical in the short term to identify the gaps in our current programing so that we can build and budget for a response that in the medium to long term is responsive to all populations.

***Example: Dearth of programme data for USDB population size estimates in South Africa.*** The dearth in programmatic data for USDB populations reflects the difficulty in estimating their respective sizes. In South Africa, estimates for FSW populations are more widely available relative to other populations at increased risk [48, 49, 50]. The most recent national estimate from 2015 estimated between 185,357 and 205,240 FSW [49] compared to the previous estimate of between 131,000 and 182,000 [50]. There are no nationally representative data on HIV service coverage for this population.

### 2.5 What to do?

If, as we have laid out above, standard approaches to estimating the data points needed for EBB will not generate accurate estimates for USDB populations, what can be done about it? In many cases, simply recognizing the limitations in each of the methods will be enough to set researchers on the right track, in terms of expanding data collection and/or revising parameters. To build a robust evidence‐based budget for USDB interventions, though, we make the following recommendations:

- Guidelines for interventions aimed at USDB populations should clearly outline the additional and/or different resources required to access and care for this population successfully, compared to the population currently reached, in order to facilitate accurate normative costing.
- Bottom‐up, micro‐costing of trial, pilot or donor‐funded programmes reaching USDB populations should clearly highlight the additional costs not typically captured in standard costs estimates of current routine programmes.
- Effectiveness of USDB interventions should not only be presented using clinical or natural outcomes; a generic health measurement such as life‐years saved, or infections averted is more likely to fully capture the benefits.
- When clinical outcomes are used for national targets (i.e. 95‐95‐95), they should be disaggregated by population to highlight any inequities and/or differing needs.
- Given the variability in population estimates, good practice guidelines need to be developed that encourage accurate, transparent estimation and adoption at national level.
- Unless it places vulnerable populations at increased risk, standards should be developed to leverage existing national surveys and databases to inform population and coverage estimates and provide critical objective evidence to document the extent of the unmet need in the marginalized.
- Application of evidence‐based budgeting methods and utilization of appropriate data sources should be documented for donors and governments funding HIV programming to highlight further gaps in existing evidence and promote transparency in decision making.

## 3 CONCLUSIONS

EBB is a strong tool to achieve the transparent and efficient allocation of resources across the different populations affected by HIV. The evidence necessary for USDB populations to be appropriately included in EBB, however, is lacking or may be biased in many SSA countries. If we do not address these deficiencies in our evidence base, the application of EBB could unfairly disadvantage these populations. Where in‐country evidence on USDB populations is sparse or missing, it would not be appropriate to use this as the reason for excluding them from the budgeting process, but rather an indication that we need to invest more to understand the needs of these underserved populations who carry a disproportionate burden.

## Competing interests

None of the authors declare any competing interests.

## Authors’ contributions

LL and GM drafted the first version of the manuscript. BL, BN, NN and SR provided critical revision.

## Abbreviations

AIDS, Acquired Immunodeficiency Syndrome; EBB, evidence‐based budgeting; HIV, human immunodeficiency virus; SSA, sub‐Saharan Africa; UNAIDS, Joint United Nations Programme on HIV/AIDS; USDB, underserved who carry a disproportionate HIV burden; WHO, World Health Organization.
